# Theoretical Insight into Antioxidant Mechanisms of Trans-Isoferulic Acid in Aqueous Medium at Different pH

**DOI:** 10.3390/ijms26125615

**Published:** 2025-06-11

**Authors:** Agnieszka Kowalska-Baron

**Affiliations:** Institute of Natural Products and Cosmetics, Faculty of Biotechnology and Food Sciences, Lodz University of Technology, Stefanowskiego 2/22, 90-537 Lodz, Poland; agnieszka.kowalska-baron@p.lodz.pl

**Keywords:** isoferulic acid, antioxidant mechanism, HT, SET, RAF, free radical scavenging, TD DFT, chelation of Fe^2+^ ions

## Abstract

This study presents the first comprehensive theoretical investigation of the antioxidant mechanisms of trans-isoferulic acid against hydroperoxyl (HOO^•^) radicals in aqueous solution, using the DFT/M062X/6-311+G(d,p)/PCM method. Thermodynamic and kinetic parameters, including reaction energy barriers and bimolecular rate constants, were determined for the three major pathways: hydrogen transfer (HT), radical adduct formation (RAF), and single electron transfer (SET). The results indicate that, at physiological pH, the RAF mechanism is both more exergonic and approximately eight-times faster than HT. At a higher pH, where the phenolate anion dominates, antioxidant activity is enhanced by an additional fast, diffusion-limited SET pathway. Isoferulic acid was also found to effectively chelate Fe^2+^ ions at pH 7.4 and above, forming stable complexes that could inhibit Fenton-type hydroxyl radical generation. Moreover, its strong UV absorption suggests a role in limiting photo-induced free radical formation. These findings not only clarify the antioxidant behavior of isoferulic acid but also provide novel theoretical insights applicable to related phenolic compounds. The compound’s multi-target antioxidant profile highlights its potential as a photoprotective agent in sunscreen formulations.

## 1. Introduction

Reactive oxygen species (ROS) belonging to a group of free radicals have an unpaired electron in the valence shell or in the excited state; thus, they are highly reactive and seek stability by accepting either a hydrogen atom or an electron. ROS are generated during essential metabolic processes in the human body (endogenous sources of ROS) and they play a very important role in the immune response and intracellular communication [1,2]. At low or moderate concentrations, ROS are substantial for human health [3,4]. However, ROS can also be derived exogenously from exposure to harmful environmental factors, such as pollution, radiation, or xenobiotics [4]. The human body possesses several defense mechanisms to neutralize ROS. However, these may be insufficient and, as a consequence, the uncontrolled accumulation of ROS may induce oxidative stress. Under oxidative stress, ROS start a pathological chain reaction, leading to the degeneration of biologically important systems such as DNA, proteins, and lipids and to the development of many illnesses such as cancer, diabetes, atherosclerosis, and neurodegenerative diseases [4,5,6]. To protect biologically important targets from free radical-mediated damage and to maintain the balance between generated and neutralized ROS, external supplements of naturally occurring antioxidants are recommended [4].

**Figure 1:**
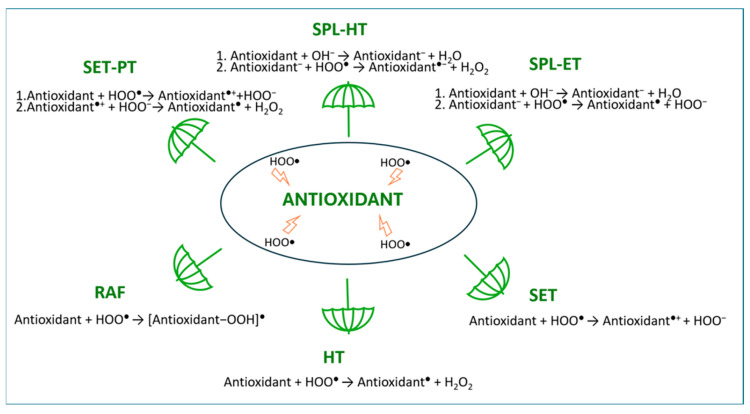
Schematic representation of mechanisms involved in primary radical scavenging.

Antioxidants are compounds that are able to decrease the level of ROS and, depending on their mechanism of action, they can be classified into three groups. The mechanism of action of type I antioxidants, called chain breakers, relies on their direct reactions with free radicals, leading to the generation of more stable and less harmful species, which terminate chain reactions. These reactions (Figure 1) usually involve a hydrogen transfer (as a radical (HT) or a proton transfer (PT)) or a single electron transfer (SET) that leads directly (HT, SET) or through a stepwise mechanism via sequential proton loss SPL (SPL-HT, SPL-ET) or SET-PT to the formation of more stable and less hazardous radical products. Such less harmful radicals may also be formed in the direct reaction between radicals and antioxidants involved in the radical adduct formation (RAF) mechanism [7,8].

The category of type II antioxidants includes (1) transition metal chelators, which prevent the Fenton reaction Fe^2+^ + H_2_O_2_ → ^•^OH + OH^−^ + Fe^3+^, thus limiting the formation of extremely reactive and harmful hydroxyl radicals [3]; and (2) chromophores absorbing in the UV range, preventing or/and reducing the UV-induced generation of various ROS, including ^•^OH radicals [9]. The latter species are the most harmful free radicals in living organisms, since they are short-lived and once formed they non-selectively attack almost any molecule in their vicinity at diffusion-limited rates, before an antioxidant can actually trap them [7,8]. It is notable that hydroxyl radical attack is considered to contribute the most to the tissue and DNA damage caused by ionizing radiation [10]. Compounds that take part in the repair of damaged biological targets of free radicals are classified into the group of type III antioxidants. Most antioxidants act via more than one of the abovementioned modes of action [8].

Hydroxycinnamic acid derivatives belong to a group of naturally occurring phytochemicals known for their antioxidant activity [11,12,13,14]. One such compound, trans-isoferulic acid ((E)-3-(3-hydroxy-4-methoxyphenyl)prop-2-enoic acid), is a phenolic secondary metabolite found in plants such as *Lobelia chinensis* [15], *Salvia miltiorrhiza* [16], and pineapple fruit [17]. This naturally occurring phytochemical has been reported to exhibit a variety of biological activities, including antioxidant [18,19,20,21,22], antiviral [23], anti-inflammatory [23], and antidiabetic effects [24]. Isoferulic acid is a derivative of hydroxycinnamic acid and an isomer of ferulic acid (4-hydroxy-3-methoxycinnamic acid), differing in the position of its functional groups: in isoferulic acid, the hydroxyl and methoxy groups are located at the meta and para positions, respectively, relative to the electron-donating –CH=CH–COOH moiety. Notably, the presence of the methoxy group (–OCH_3_) on the aromatic ring influences the free radical scavenging activity of hydroxycinnamic acid derivatives [18].

Although the antioxidant ability of isoferulic acid [18,19] and its complexes with Mg(II) and Mn(II)/Na(I) cations [18] has been explored from an experimental point of view, to the best of my knowledge there is a lack of theoretical studies devoted to the mechanisms of free radical scavenging of isoferulic acid in aqueous medium. In particular, so far there are no theoretical reports regarding the kinetic aspects of the free radical scavenging mechanism of isoferulic acid against HOO^●^ radicals. Thermodynamic data are often insufficient in unravelling the free radical scavenging mechanism, since exergonic reactions can occur at different rates, either slow or fast, and on the other hand moderately endergonic processes (with ΔG < 10 kcal/mol), especially those involved in SET and RAF reactions, may still be relevant to the overall antioxidant capacity [6,7,8,25].

The aim of this work is to contribute to a deeper understanding of the antioxidant activity of isoferulic acid by performing a comprehensive theoretical investigation of all possible type I antioxidant mechanisms, including hydrogen transfer (HT), radical adduct formation (RAF), single electron transfer (SET), sequential proton loss electron transfer (SPL-ET), SET–proton transfer (SET-PT), and SPL–HT, in the presence of hydroperoxyl radicals (HOO^•^), using the M062X/6-311+G(d,p)/PCM level of theory. Hydroperoxyl radicals, formed by the protonation of superoxide (a common intermediate in biochemical processes [10]), are known to contribute to tumor development and initiate lipid peroxidation. Due to their moderate reactivity and longer half-life compared to hydroxyl radicals, HOO^•^ radicals are widely recommended as target species in theoretical antioxidant studies [6,7,8]. It is worth noting that the deprotonated form, O_2_^•−^, which is present at physiological pH, is significantly less reactive and does not pose a comparable oxidative risk [8].

The theoretical approach applied in this study—M062X/6-311+G(d,p)—is recognized as one of the most reliable methods for studying the thermodynamics and kinetics of radical-involved reactions [6,7,25], offering dependable predictions of reactivity and energetics [26,27,28]. The investigation began with an in-depth analysis of the electronic structure of isoferulic acid (indices related to Frontier Molecular Orbitals Theory were evaluated, and the most reactive sites for radical attack were indicated) and its reactivity (based on the thermochemical intrinsic reactivity indices). Then, the thermodynamic feasibility of all possible primary antioxidant pathways was evaluated in terms of the M062X/6-311+G(d,p)/PCM-calculated Gibbs free energies of reactions involved in the HT, RAF, SET, SPL-ET, SPL-HT, and SET-PT mechanisms (with explicit incorporation of HOO^•^ radicals). Next, for the exergonic and isoergonic paths (RAF and HT, SET) kinetic calculations were performed to determine reaction energy barriers and bimolecular rate constants. Based on the obtained thermodynamic and kinetic data, the preferred mechanisms of HOO^•^ scavenging by isoferulic acid as an antioxidant of type I in aqueous medium of different pH were identified.

It is important to emphasize that despite the availability of various experimental assays for measuring antioxidant capacity—such as DPPH^•^ (1,1-diphenyl-2-picrylhydrazyl radical), ABTS^●+^ (3-ethylbenzthiazoline-6-sulfonic acid diammonium salt), and CUPRAC (cupric ions reducing power)—there is currently no universal method capable of quantitatively and accurately assessing antioxidant activity. This is due to differences in the solvent systems, pH conditions, reaction times, and the mechanisms underlying each assay. For example, both the SET and HT mechanisms contribute to the antioxidant response in ABTS^•+^ and DPPH^•^ assays. Additionally, experimental results are often expressed in non-standardized units (e.g., Trolox equivalents, IC_50_), which complicates meaningful comparisons between studies or with theoretical data [25].

Therefore, selecting an appropriate experimental assay should be guided by prior knowledge of the dominant antioxidant mechanism of a particular compound. In this context, theoretical studies—such as the present work—are invaluable. Recent advances in computational chemistry and computer technology have established theoretical methods as reliable and insightful tools for elucidating thermodynamic and kinetic aspects of antioxidant behavior. Notably, theoretical kinetic approaches allow for the incorporation of critical effects such as quantum tunneling and pH-dependence, which are often difficult to evaluate experimentally [6,25].

To complement the type I mechanism analysis, the type II antioxidant potential of isoferulic acid was also explored. Its ability to chelate Fe^2+^ ions—key catalysts in hydroxyl radical generation via the Fenton reaction—was evaluated, as well as its capacity to absorb UVB-range electromagnetic radiation, which may contribute to reducing photo-induced oxidative stress. The absorption spectra of all acid–base species of isoferulic acid in aqueous medium (together with the spectroscopic parameters of the electronic transitions that define main absorption bands) were predicted by applying the TD DFT(M062X)/6-311+G(d,p)/PCM method.

The obtained results showed that isoferulic acid exhibits antioxidant activity via multiple mechanisms: the antioxidant mode of action of type I against HOO^•^ radicals (within the RAF and HT pathways, and additionally via the SET mechanism in basic medium) and via the chelation of catalytic Fe^2+^ ions suppressing their involvement in the Fenton reaction that generates harmful ^•^OH radicals. Moreover, isoferulic acid has the ability to absorb electromagnetic radiation within the UVB wavelength range, thus offering additional protection against photo-induced oxidative stress. The novel theoretical insights presented in this study not only clarify the multi-target antioxidant potential of isoferulic acid—underscoring its possible utility as a photoprotective agent in sunscreen formulations—but also extend our understanding of free radical scavenging mechanisms by structurally related phenolic compounds.

## 2. Results and Discussion

### 2.1. Theoretical Determination of Acid–Base Equilibria of Isoferulic Acid (pK_a_ Values of Isoferulic Acid)

**Figure 2:**
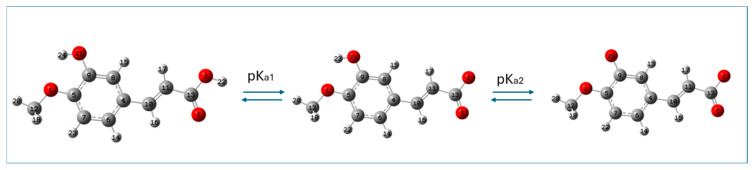
M062X/6-311+G(d,p)/PCM(water)-optimized (neutral and deprotonated) forms of isoferulic acid. Atomic labels according to Gaussian 09.

Isoferulic acid has an ionizable carboxylic moiety and hydroxyl group and is weakly acidic; therefore, depending on the pH value of the aqueous medium, three species of this compound are possible: neutral, monoanionic, and dianionic. The M062X/6-311+G(d,p)/PCM(water)-optimized structures of neutral and deprotonated species of isoferulic acid are shown in Figure 2.

The relative abundance of species with acid–base equilibria in aqueous medium depends on their pK_a_ values. The experimental pK_a_ values of isoferulic acid have not yet been determined; however, according to [29], the predicted pK_a1_ value of isoferulic acid is 4.53 ± 0.11. The theoretically predicted values of pK_a_ for isoferulic acid determined with the use of the isodesmic method and the parameter-fitting method are gathered in Table 1.

From Table 1, it may be seen that isodesmic method slightly underestimates the pK_a1_ value (the discrepancy between the predicted pK_a1_ taken from the literature (4.53) and the calculated value is 0.43 pK_a_ unit), while the value determined by applying the parameter-fitting method is overestimated (of 0.77 pK_a_ unit). Since it is more difficult to detach a proton from the ion than from the neutral species, pK_a2_ should be increased in comparison with pK_a1_.

The difference between the pK_a2_ and pK_a1_ values determined using the isodesmic method is 2.2, whereas for the parameter-fitting method, this difference is 5.5 pK_a_ units. The latter value is closer to the differences observed experimentally for structurally related compounds. For instance, the pK_a2_–pK_a1_ difference for isovanillic acid is 5.2 [30], while for ferulic acid it is approximately 4.1 [30] or 4.5 pK_a_ units [31]. These comparisons suggest that the parameter-fitting method yields more realistic estimates of the second pK_a_ value for isoferulic acid. Therefore, the pK_a_ values of isoferulic acid determined with the use of the parameter-fitting method seem to be more reliable and will be used in this study to estimate the molar fractions of the acid–base forms of isoferulic acid at different pH values.

At physiological pH (7.4), the monoanionic form of isoferulic acid prevails (99.17%), the fraction of the neutral form is 0.79%, and the fraction of the dianionic form is 0.04%. At high pH values (pH > pK_a2_), the dianionic form of isoferulic acid starts to prevail over the monoanionic form (at pH 10, dianionic forms are dominant (61%) over monoanionic forms (39%), while neutral forms are practically absent). In the dianionic forms of isoferulic acid, carboxylic and hydroxyl groups are deprotonated, making hydrogen donation routes inaccessible, but these forms may still exhibit antioxidant activity through the electron-related channel.

At pH < pK_a1_, neutral species of isoferulic acid prevail over monoanions (the lower the pH value, the fewer monoanionic forms of the compound present), while dianionic species are practically absent. The presence of a hydroxyl group in neutral forms offers a hydrogen-related channel in the free radical scavenging mechanism.

Since (1) the pH of the medium has been previously reported to affect the antioxidant activity of hydroxycinnamic acid derivatives [6,7,11,12,14] and (2) electron-related channels incorporating deprotonated species have been shown to proceed with diffusion-limited rate constants [11,12], all acid–base species of isoferulic acid will be considered in this study.

### 2.2. Prediction of Antioxidant Properties of Isoferulic Acid Based on the Indices Related to Frontier Molecular Orbitals Theory

The determined indices related to Frontier Molecular Orbitals Theory—electronegativity (χ), chemical potential (μ), global hardness (η), global softness (S), and electrophilicity (ω) together with electron-donating power (ω−) and electron-accepting power (ω+)—are gathered in Table 2.

From Table 2, it may be seen that deprotonation of isoferulic acid leads to a decrease in ionization potential (IP), electrophilicity (ω), electronegativity (χ), electron-donating power (ω−), and electron-accepting power (ω+). The lower the IP, χ, ω, and ω− values, the more likely it is that an antioxidant molecule will act as an electron donor in interactions with radical species. The lower the value of the electron-accepting power (ω+), global softness (S), and global hardness (η) and electron affinity (EA), the more unlikely it is that the antioxidant molecule will accept electrons in interactions with other species. The calculated indices (IP, ω, χ, ω−, ω+, EA) show that each subsequent deprotonation increases the electron-donating properties of isoferulic acid. It should be noted that the applied approximations of orbitals as eigenvalues of ionization potential or electron affinity neglect electron correlation and depend on the method and basis set employed in their calculations [8,31]. This may lead to inaccurate values and thus to invalid conclusions about the electrophilic character of the species [8].

### 2.3. Prediction of Radical Attack Site

**Figure 3:**
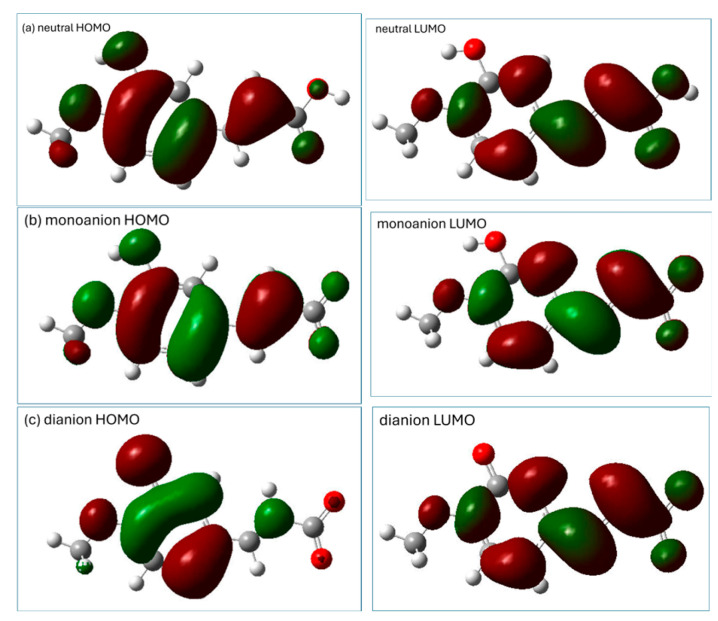
Visualization of HOMO and LUMO (calculated using M062X/6-311+G(d,p)/PCM(water) method) of isoferulic acid: (**a**) neutral form, (**b**) monoanion, (**c**) dianion.

The distribution of the HOMO (the highest-occupied molecular orbital) and LUMO (the lowest-unoccupied molecular orbital) may provide preliminary information which may be helpful in the prediction of potential sites for radical attack [8]. A representation of the HOMO and LUMO orbitals of the three studied forms of isoferulic acid (calculated using the M062X/6-311+G(d,p)/PCM(water) method) is shown in Figure 3.

The distribution of electronic density in the HOMO (Figure 3) indicates that for all the studied species, π electrons of the aromatic ring and the C_11_ carbon atom of the -C=C- chain may be important in electron-donating routes of the free radical scavenging mechanism. The phenolic group of the neutral and monoanionic forms of isoferulic acid offers the most probable site involved within the HT mechanism. However, based on the visualization of Frontier orbitals itself, it is hard to indicate unambiguously the most probable radical attack site, especially that involved in the RAF mechanism engaging dianionic species. Condensed Fukui functions for radical attack $f_{A}^{0}$ calculated for an arbitrary atom A offer a more convenient and straightforward way to predict the reactive sites of reactions with free radicals, since $f_{A}^{0}$ values depend only on the atom’s electronegativity and electron density location [8]. Generally, the higher the $f_{A}^{0}$ value for a particular atom, the more prone that atom is to radical attack. The condensed Fukui functions for radical attack $f_{A}^{0}$ for C and O atoms of dianionic species of isoferulic acid (Table 3) indicate that among carbon atoms, the C_11_ of the conjugated carbon chain -C_10_=C_11_- is the most probable site for radical attack.

The negative values of condensed Fukui functions for C_12_ and C_9_ (see Table 3) may be explained as a result of the small interatomic distances [8,32] and orbital expansion and contraction [33]. The condensed Fukui functions can be used to predict the possible radical attack sites in a very straightforward way, but they do not provide information regarding the mechanism involved in the radical attack. However, this information can be gained from the calculated intrinsic thermochemical parameters.

### 2.4. Evaluation of Free Radical Scavenging Ability via Intrinsic Thermochemical Parameters

Intrinsic thermochemical reactivity indices can give important preliminary data that will be helpful for evaluating the preferred antioxidant reaction pathways and showing the direction of further research [8]. For example, bond dissociation enthalpy (BDE), adiabatic ionization potential (AIP), and proton affinity (PA) are considered as the primary indices of the HT, SET, and SPL mechanisms, respectively. M062X/6-311+G(d,p)/PCM(water)-calculated values of AIP, BDE, PA, and proton dissociation enthalpy (PDE) are gathered in Table 4.

In calculations of indices involving the detachment of protons, the sequence of deprotonation was the same as in pK_a_ determination (carboxylic moiety, phenolic group). From the calculated BDE values collected in Table 4, it may be seen that for neutral and monoanionic forms of isoferulic acid the HT pathway may be considered as a probable mode of antioxidant action.

**Figure 4:**
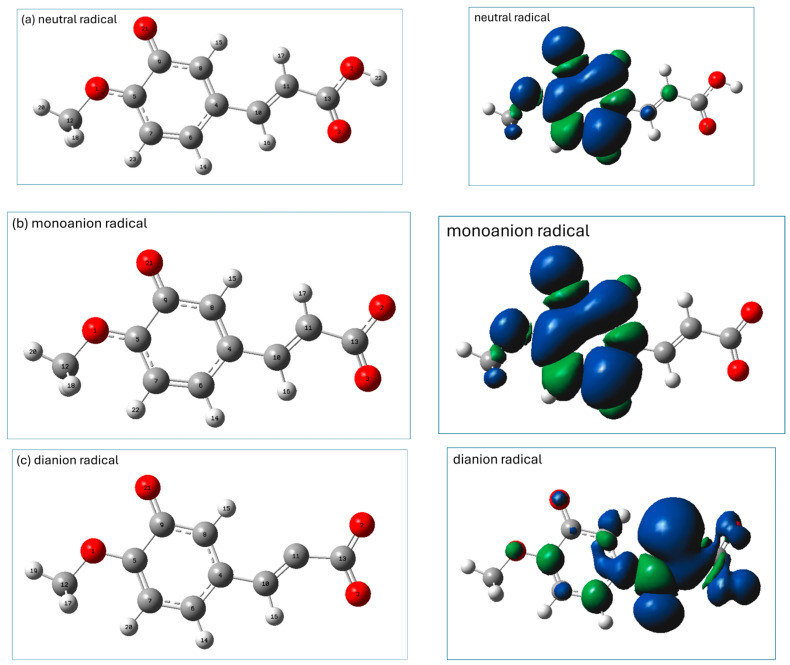
M062X/6-311+G(d,p)/PCM(water)-calculated geometries (left panel) of the most stable antioxidant radicals of (**a**) neutral, (**b**) monoanionic, and (**c**) dianionic species of isoferulic acid formed in the HT mechanism, together with their spin density distributions (right panel). Atomic labels according to Gaussian 09.

Graphical presentations of the most stable radicals of the neutral, monoanionic, and dianionic forms of isoferulic acid formed within the HT antioxidant mechanism (calculated at the M062X/6-311+(d,p)/PCM(water) level) are shown in Figure 4, together with their spin density distributions.

The most stable radicals of neutral and monoanionic forms of isoferulic acid arise from the homolytic breakage of the phenolic group. In the spin density representation of these radicals (Figure 4a,b), the unpaired electron is delocalized over the entire aromatic ring and methoxy group.

In the dianionic form of isoferulic acid there is no OH group, and the most stable radical in the HT mechanism is that formed via H subtraction from the C_11_H_17_ group_._ However, the BDE value for the subtraction of H from C_11_H is considerably higher than that for the other forms of isoferulic acid, which suggests that the HT mechanism is unlikely for the dianionic form of the studied compound. As can be seen from the spin density distribution (see Figure 4c, right panel), the unpaired electron is mainly delocalized over a vinyl bond and COO^−^ group (which favors resonance and conjugation effects) and also, but to a lesser extent, on the carbon atoms of the aromatic ring and methoxy group, suggesting significant stabilization of the radical structure.

The proton-donating ability of isoferulic acid is characterized by the calculated value of PA. For neutral and monoanionic forms of isoferulic acid, PA values are lower than BDE values (Table 4), suggesting that these forms are more prone to undergoing the SPL mechanism, which may be followed by the ET or HT mechanism. From Table 4, it may be seen that both PA and PDE values increase with deprotonation, indicating that with each subsequent deprotonation, routes that includes proton donation become more energetically demanding.

AIP is characteristic of the SET mechanism and is defined as the minimum energy required to transfer an electron from the antioxidant to the free radical to form a cationic radical species. The lower the AIP value, the more easily the electron is transferred and the higher the antioxidant activity via the SET mechanism. From Table 4, it may be noticed that the AIP values decrease with deprotonation, indicating that the increase in the pH of the medium favors electron-related channels (the SET pathway). After the first deprotonation (of the carboxylic group), the AIP value is decreased by only 6%, which suggests that the electron-donating ability of monoanionic forms may be not sufficient to promote a fast electron-transfer reaction. However, the second deprotonation resulting in the formation of phenolate dianions leads to a 30% decrease in AIP; thus, dianionic forms of isoferulic acid are expected to be good electron donors in the SET mechanism. A necessary condition for the SET reaction is that the EA of a free radical is higher than the AIP of the antioxidant [6,8]. The EA of the free radical R^●^ is the enthalpy change accompanying the reaction R^●^ + e^−^ = R^−^ [34]. The M062X/6-311+G(d,p)/PCM(water)-calculated values of EA for HOO^●^ and other selected radicals are gathered in Table 5.

The thermochemical data collected in Table 4 and Table 5 suggest that dianionic forms of isoferulic acid may react via the SET mechanism with all the tested radicals with the exception of the HOO^●^ radical, since only for the dianionic form reacting with the HOO^●^ radical is the energy released by the free radical on gaining the electron not sufficient to compensate for the dianionic AIP. However, it should be noted that in thermochemical EA calculation, the enthalpy of the electron is required, which cannot be estimated directly; therefore, it is necessary to use one of the widely varying reference values [8]. It is worthy of note that the energy released by the Cl_3_CO^•^ radical after accepting an electron is enough to compensate for the AIP of both the neutral and deprotonated forms of isoferulic acid; thus, all the acid–base species may potentially react via the SET mechanism with Cl_3_CO^•^ radicals. This clearly demonstrates that while investigating antioxidant mechanisms, not only should the antioxidant itself be considered but also the radical with which it interacts.

### 2.5. Possible Pathways of HOO^●^ Free Radical Scavenging by Isoferulic Acid

The Gibbs free energies associated with the reactions involving isoferulic acid and HOO^●^ radicals related to the HT, RAF, SET, SET-PT, SPL-ET, and SPL-HT mechanisms (Table 6) give more relevant data concerning the antioxidant mechanism than intrinsic thermochemical reactivity indices alone.

When considering the RAF mechanism, the reactive site at C_11_ of the -C=C-COO^−^(H^+^) conjugated chain for radical addition was taken into account. The calculated ΔG of the reaction involved in the RAF pathway showed that for the neutral form of isoferulic acid the reaction is moderately endergonic, but deprotonation favors the formation of radical adducts at C_11_ (as for monoanionic and dianionic forms of isoferulic acid, the RAF(C_11_) reactions are exergonic; see Table 6).

**Figure 5:**
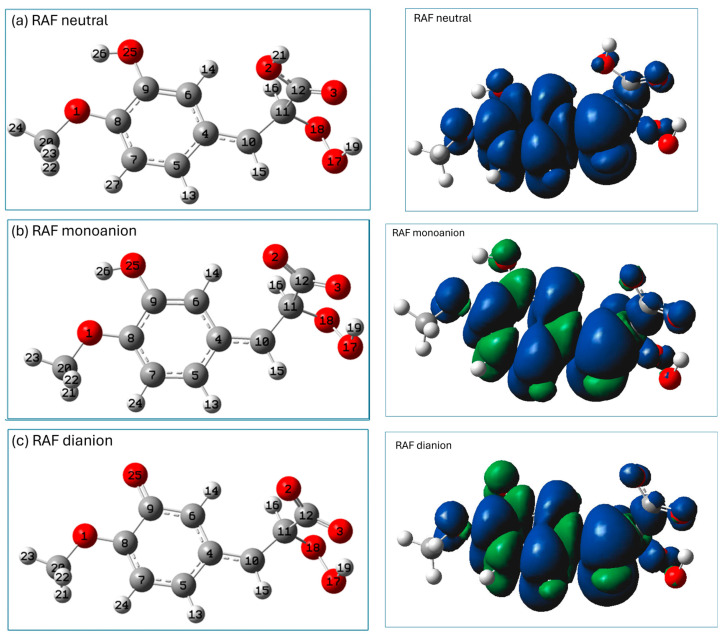
M062X/6-311G+(d,p)/PCM(water)-calculated geometries (left panel) of the most stable products formed within the RAF-C_11_ mechanism incorporating (**a**) neutral, (**b**) monoanionic, and (**c**) dianionic species of isoferulic acid together with their spin density distributions (right panel). Atomic labels according to Gaussian 09.

The geometries of radical adducts formed as a result of HOO^●^ addition to C_11_ of the neutral and deprotonated forms of isoferulic acid are presented in Figure 5, together with their spin density distributions. Inspection of the geometrical parameters of these adducts (gathered in Table 7) reveals that the RAF products generally show similar geometrical features: the formation of radical adducts involves a particular rearrangement of the carboxylic or carboxylate group (which in the final product is perpendicular to the aromatic ring) to facilitate the hydrogen-bonding interaction of its carbonyl oxygen with the attached radical (see Table 7; the geometrical parameters of these hydrogen-bonded interactions are marked with bold font). Moreover, the deprotonation of the carboxylic group increases the strength of this hydrogen-bonding interaction (as indicated by the shortening of the O_3_H_19_ distance and the increase in the linearity of the hydrogen bond (see the O_17_H_19_O_3_ angle, Table 7)). In the RAF products, the C_4_C_10_ bond, which is in the nearby surroundings of the aromatic ring, is shorter than in the parent molecules, and the C_4_C_10_C_11_ bond angle is 125°, while that of C_10_C_11_C_12_ is around 112°. Moreover, in the RAF (C_11_) adducts, the acyclic carbon chain is almost linear with an aromatic ring (see the values of C_6_C_4_C_10_C_11_ in Table 7), which favors resonance and delocalization effects. As the result, the unpaired electron originated through the reaction with the free radicals has the possibility to be spread over the entire molecule, resulting in a significant radical adduct stabilization (see spin density distribution in Figure 5, right panel).

From Table 6, it may be seen that the reaction involved in the HT mechanism is exergonic with neutral species of isoferulic acid when H is subtracted from the phenolic group. Deprotonation of the COOH group makes hydrogen subtraction from the phenolic group easier, as it leads to a decrease in the Gibbs free energy of the reaction involved in the HT mechanism (see Table 6). It should be noted that in the case of monoanionic and dianionic forms of isoferulic acid, the Gibbs free energy of HT is equivalent (identical) to the second step of the SPL-HT mechanism (see Table 6). In the case of the dianionic form of isoferulic acid, the reaction of hydrogen atom subtraction from C_11_ is endergonic (>10 kcal/mol); therefore, at high pH values, the HT mechanism leading to the formation of this kind of radical of the compound under study is unlikely.

The Gibbs energy of the reaction involved in the SET mechanism decreases with the ionic character of the antioxidant and becomes moderately endergonic for the dianionic form of isoferulic acid, indicating the SET mechanism as a feasible mode of free radical scavenging activity for these species. For neutral and monoanionic forms of isoferulic acid, the SET mechanism is not thermodynamically favorable (as the formation of radical cations is a highly endergonic process). It should be mentioned that in the case of ionic forms of isoferulic acid, the Gibbs free energy of SET is the same as the second step of the SPL-ET mechanism (see Table 6).

Both the SPL-HAT and SPL-ET mechanisms are initiated by proton dissociation, the proclivity of which is controlled by a medium pH and acid–base equilibrium. The results of calculations showed that in the presence of a base (OH^−^), the proton dissociation is highly exergonic. The second dissociation is more energetically demanding (less exergonic) due to the increasing negative charge of the initial structure, which makes proton detachment more difficult.

Since (1) the first step of the SET-PT mechanism is highly endergonic for neutral and monoanionic species of isoferulic acid, and (2) the second step of this mechanism is endergonic (>10 kcal/mol) for the dianionic form of the studied compound, SET-PT may be considered as an unfavorable pathway of free radical scavenging by the compound under study.

Thermodynamics is used to predict the direction of spontaneous chemical reactions at equilibrium, but it says nothing about how quickly the reaction approaches equilibrium. An exergonic reaction can occur at either fast or slow rates [6,7,8]. On the other hand, moderately endergonic reactions (with ΔG less than 10 kcal/mol), particularly within RAF and SET mechanisms, may still contribute to antioxidant activity [6,7,8]. Moreover, for SET reactions, thermochemical and kinetic data may show opposing trends [6,7,8,35].

Additionally, quantum mechanical phenomena like tunneling should be considered for reactions involving the transference of small mass particles (hydrogen abstraction reactions) or with reactions with narrow barriers [6]. Such processes include the HT reaction as well as some RAF pathways [6,7,8].

The reaction between the neutral form of isoferulic acid and the HOO^●^ radical within the RAF mechanism and the reaction involved in the SET mechanism with dianionic species of the compound under study are endergonic, but lower than the recommended threshold of 10 kcal/mol [6,7,25], implying that these routes may still contribute to antioxidant activity. The Gibbs free energies of the other endergonic reactions are significantly higher than the mentioned threshold. These reactions are thermodynamically unfavorable and kinetic calculations will not be performed for them.

As mentioned in Section 2.1, at a physiological pH value, the monoanionic forms of isoferulic acid prevail (99.17%), while the neutral and dianionic forms constitute only 0.79% and 0.04% of the acid–base species population. The Gibbs free energy values of the reactions between the neutral (and dianionic) forms of isoferulic acid and HOO^●^ radicals involved in the RAF mechanism are higher as compared to those engaging monoanionic species. Additionally, the HT reaction involving neutral species of isoferulic acid is less exergonic than that with monoanionic forms of the compound under study. It has been evidenced that the Gibbs free energy of the reaction involved in the HT mechanism is proportional to the activation energy of this reaction, and hence the reaction rate of this mechanism [8,36]. Therefore, it may be expected that HT reaction with neutral species and RAF reactions involving neutral and dianionic forms of isoferulic acid will proceed at a lower rate as compared to those incorporating monoanionic species.

Taking into account the above considerations, the kinetic aspects of the reactions between monoanionic forms of isoferulic acid and HOO^●^ radicals within the RAF and HT mechanisms including tunneling phenomena should be addressed. Moreover, since the SET reaction involving dianionic species is moderately endergonic (less than 10 kcal/mol [6,7]), the assumptions deduced from the thermodynamic consideration of this reaction also need to be completed with kinetic investigations.

### 2.6. Kinetics of Reactions Involved in the HT, SET, and RAF Mechanisms

In contrast to the SET mechanism, HT and RAF reactions proceed through a transition state, TS. Therefore, stationary points along the reaction pathways within the HT and RAF mechanisms have to be carefully investigated.

**Figure 6:**
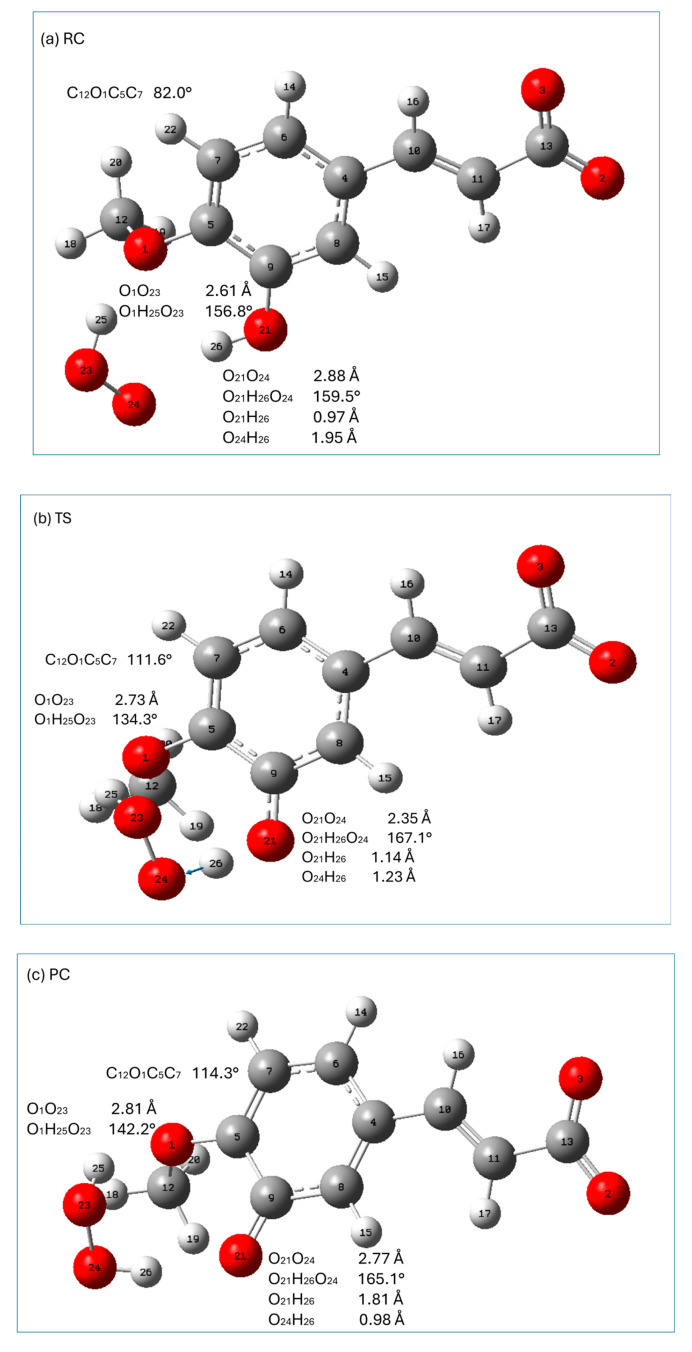
Graphical presentations of the stationary points ((**a**) reactant complex (RC), (**b**) transition state (TS), and (**c**) product complex (PC)) encountered along the HT reaction pathway of isoferulic acid (monoanionic form) with hydroperoxyl radicals. Displacement vectors of imaginary frequency at 3042.07*i* cm^−1^ are shown as blue arrows. Atomic labels according to Gaussian 09.

**Figure 7:**
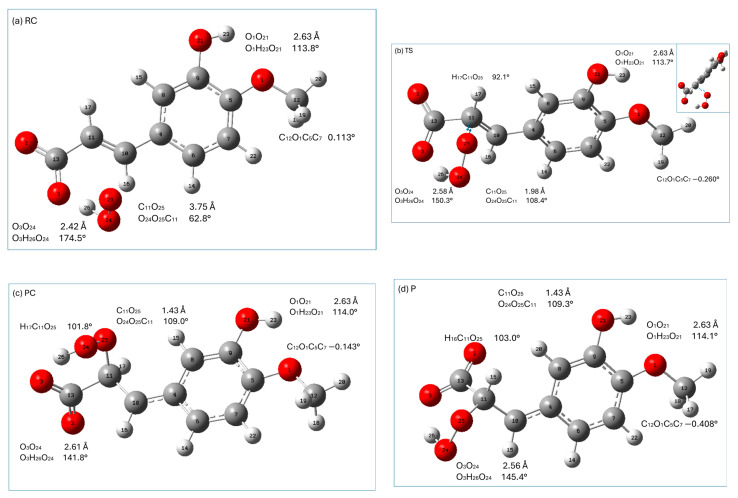
Graphical presentations of the stationary points ((**a**) reactant complex (RC), (**b**) transition state (TS), (**c**) product complex (PC), and (**d**) product (P)) encountered along the RAF (C_11_) reaction pathway of isoferulic acid (monoanionic form) with hydroperoxyl radicals. Displacement vectors of imaginary frequency at 719.01*i* cm^−1^ are shown as blue arrows. Atomic labels according to Gaussian 09.

Graphical presentations of the stationary points (reactant complex (RC), transition state (TS), and product complex (PC)) along the HT reaction pathway of the monoanionic form of isoferulic acid with hydroperoxyl radicals are presented in Figure 6, whereas those encountered in the RAF mechanism are shown in Figure 7. The relative enthalpies and Gibbs free energies of the stationary points encountered along the reaction pathways within the HT and RAF mechanisms with respect to the reactants are gathered in Table 8.

All HT stationary points are characterized by a significant structural stabilization resulting from the presence of an intermolecular hydrogen-bonding interaction between the O_21_H_26_ group of isoferulic acid and the O_24_ oxygen atom of the free radical (RC) and the O_24_H_26_ group of hydrogen peroxide and the O_21_ oxygen atom of isoferulic acid (PC). Geometrical parameters of these interactions indicate that they are strong (the distance between the electronegative atoms taking part in these interactions is less than 3 Å and they are nearly linear; see Figure 6). In particular, in TS the distance between the electronegative atoms taking part in the intermolecular interaction O_21_O_24_ is 2.35 Å, and the angle between the atoms incorporated in the interaction is 167.1°, which is a favorable geometrical rearrangement between atoms for the HT reaction. Additional stabilization of all HT stationary points arises from the presence of a hydrogen-bonding interaction between the methoxy group of isoferulic acid and the H_25_O_23_ group of the HOO^●^ radical (RC, TS) or H_2_O_2_ (PC).

The hydrogen abstraction reaction within the HT mechanism passes through a TS (imaginary frequency 3042.07*i* cm^−1^ corresponding to the stretching of the O_21_H_26_, bond) in which the H_26_ atom attached to O_21_ is transferred to the radical (it is indicated by the change in the H_26_O_24_ distance from 1.95 Å in RC, 1.23Å in TS, and 0.98 Å in PC; see Figure 6).

From Table 8, it may be noticed that in the case of the HT mechanism all stationary points with the exception of TS have a lower enthalpy than the reactants. The reaction involved in the HT mechanism (leading to the formation of products) is exothermic and exergonic. The free Gibbs energy of RC, TS, and PC is higher than that of the reagents. This is most probably due to the decrease in entropy related to the reduction in the degrees of freedom in the system caused by directional and ordered hydrogen-bonding interactions. Both relative enthalpy and free energy increase from RC up to TS and then decrease down to the product complex. When going from PC to the separated products, the Gibbs energy continues to decrease, which can be related to the positive entropy change of the separated products: hydrogen peroxide (H_2_O_2_) molecules and antioxidant radicals (the reduced amount of order and the increased degrees of freedom of the separated products).

In the RAF reaction pathway, the HOO^●^ radical approaches isoferulic acid at a C_11_O_25_ distance of 3.75 Å (O_24_O_25_C_11_ is 62.8°; see Figure 7a), forming a strong (O_3_O_24_ distance is 2.42 Å; O_3_H_26_O_24_ angle is 174.5°) hydrogen-bonding interaction with a carboxyl moiety and weakly interacting with the π electrons of C_11_ of the double-bonded carbon chain (-C=C-). The hydroperoxyl radical is attached to C_11_ through the TS state, the nature of which was confirmed by the presence of one imaginary frequency at 719.01*i* cm^−1^ corresponding to the stretching of the C_11_-O_25_ bond. In TS, C_11_ retains the geometrical features of carbon sp^2^ hybridization (H_17_C_11_C_10_ is 120.2°; C_10_C_11_ is 1.37 Å) and the conjugated carbon chain is nearly planar (C_4_C_10_C_11_C_13_ is 167.1°). In TS, the distance between C_11_O_25_ is shortened from 3.75Å (RC) to 1.98 Å, and the angle O_24_O_25_C_11_ increased from 62.8° in RC to 108.4°. TS evolves into PC, in which the C_11_O_25_ bond is completely formed (C_11_O_25_ 1.43 Å; O_24_O_25_C_11_ 109.0°). It should be noticed that in TS, the hydrogen-bonding interaction between the carboxyl group and the radical is attenuated (as indicated by the increase in the O_3_O_24_ distance and distortion from linearity of the O_3_H_26_O_24_ angle). In PC and the final RAF product, the abovementioned hydrogen bond is slightly weakened as compared to that of TS. This is caused by a slight rotation of the carboxylic group around C_11_C_13_ bond to facilitate a weak intermolecular interaction (C_11_H_16_O_2_ is ~65°; C_11_O_2_ is ~2.4 Å) of the O_2_ carboxyl oxygen with the C_11_H_16_ group. It should be noted that all stationary points and the final product of the RAF reaction between isoferulic acid and the hydroperoxyl radical are characterized by a weak intramolecular hydrogen-bonding interaction between the methoxy group (which is coplanar with the aromatic ring) and the phenolic group of the compound under study (the O_1_O_21_ distance assumes the value of 2.63 Å; O_1_H_23_O_21_ is 114°; Figure 7) contributing to the stabilization of the interacting systems.

All stationary points along the RAF pathway with the exception of TS have a lower enthalpy than the reactants (Table 8). The enthalpy increases when going from RC to TS, and then decreases, and the overall reaction leading to the formation of PC and P is exothermic. The Gibbs free energy increases up to TS (which is related to the decrease in entropy caused by the reduced degree of freedom in the molecular system), and then decreases when the PC and final product are formed.

Kinetic parameters of the exergonic reactions involved in the HT and RAF antioxidant mechanisms of monoanionic species of isoferulic acid towards HOO^●^ are gathered in Table 9, together with a temperature-dependent transmission coefficient accounting for quantum tunneling effects by employing the Eckart barrier. The kinetic data for the moderately endergonic SET reaction involving dianionic forms of isoferulic acid and HOO^●^ are presented in Table 10.

**Figure 8:**
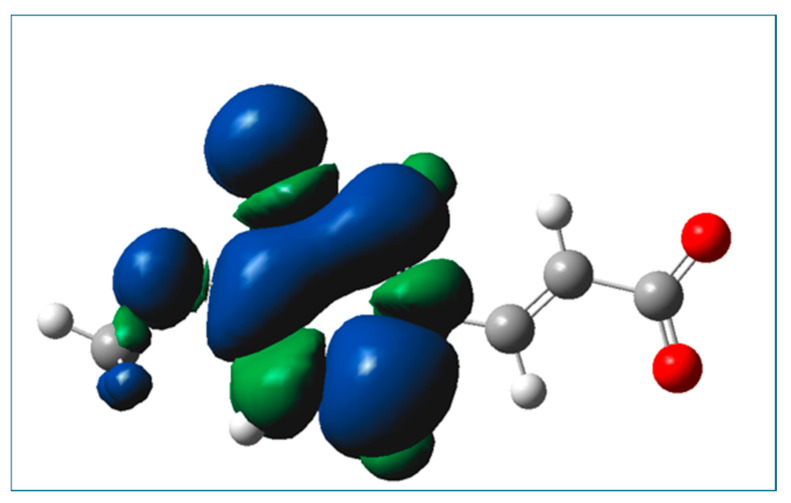
Spin density of the resultant radical cation formed in the SET mechanism involving the dianionic form of isoferulic acid.

The determined kinetic data (see k_app_, Table 9) indicate that the RAF reaction incorporating monoanionic forms of isoferulic acid and HOO^●^ radicals proceeds eight-times faster as compared to the HT reactions involving these species. Based on the values of Gibbs energy for RAF reactions (see Table 6), and assuming that the reaction’s Gibbs free energy is proportional to the rate constant of the reactions, that is, the more exergonic the reaction, the faster the reaction, it is reasonable to expect that in the case of the dianionic form of isoferulic acid, the RAF reaction would proceed with a comparable rate constant to that for RAF incorporating monoanionic species. Comparing the calculated values of the diffusion-corrected apparent rate constants (Table 10) with data from Table 9, it may be noticed that the SET reaction involving dianionic species of the compound under study and HOO^●^ radicals is almost 14,000-times faster as compared to that within the HT mechanism incorporating monoanionic species. The spin density of the resultant radical formed in the SET mechanism involving the dianionic form of isoferulic acid is presented in Figure 8, from which it may be seen that the radical is a planar species and the unpaired electron is mainly spread over the aromatic ring.

In the literature, there is a lack of both experimental and theoretical kinetic data on the reactions involved in the primary antioxidant mechanisms of isoferulic acid, which limits the possibility of directly comparing the results obtained in this study. However, relevant data are available for its isomer, ferulic acid. For instance, Carmona-Galano et al. [11], using the M052X/6-311+G(d,p)/SMD method, reported the following rate constants for the reaction of ferulic acid with HOO^•^ radicals: SET—3.36 × 10^8^ M^−1^s^−1^, HT—2.30 × 10^4^ M^−1^s^−1^, and RAF—8.05 × 10^2^ M^−1^s^−1^ [11].

It should be noted that the computed apparent rate constants for the RAF, HT, and SET reactions with hydroperoxyl radicals (HOO^•^) were calculated without accounting for the actual molar fraction of HOO^•^ at physiological pH, which should be taken into account when comparing theoretical overall rate constants with experimentally derived ones [25].

Having the optimized structure of dianionic radical cation species of isoferulic acid, it is tempting to calculate kinetic parameters for SET reactions involving a larger set of free radicals (Table 10). The determined kinetic data revealed that these reactions are characterized by a very low energy barrier (from 0.2 kcal/mol in the case of highly reactive hydroxyl radicals to 4.92 kcal/mol for HOO^●^ radicals), and for all the radicals tested the determined bimolecular rate constants of the reactions within the SET mechanism are very high, above the diffusion limit (above 2 × 10^8^ M^−1^s^−1^) [8]. The differences in reactivity of the dianionic species of isoferulic acid toward the tested radicals in the SET mechanism can be explained by variations in the radicals’ electron-acceptor character [11]. From Table 10, it may be seen that the absolute values of the Gibbs free energy of the reactions involved in the SET mechanism are lower than the corresponding reorganization energies, indicating that the reactions are not in the inverted region of the Marcus parabola, where the reaction barrier increases as ΔG decreases [6,25,37].

The data collected in Table 10 show that the barrier for the SET pathway with hydroxyl radials is very low (below 1 kJ/mol). Previous studies have shown that reactions between hydroxycinnamic acids and HO^●^ proceed very quickly whatever the path followed in the antioxidant mode of action [3,25,38]. Since hydroxyl radicals are highly reactive and not selective due to their propensity to react with almost any molecule in their proximity, including the most important biological macromolecules, there is a need to limit the formation of these species [25], and any process that limits their formation is important. One of these processes, which indirectly limits the generation of these radicals, is the chelation of Fe^2+^ ions, thus suppressing their involvement in the Fenton reaction.

### 2.7. Fe^2+^ Ions Chelating Properties of Isoferulic Acid

**Figure 9:**
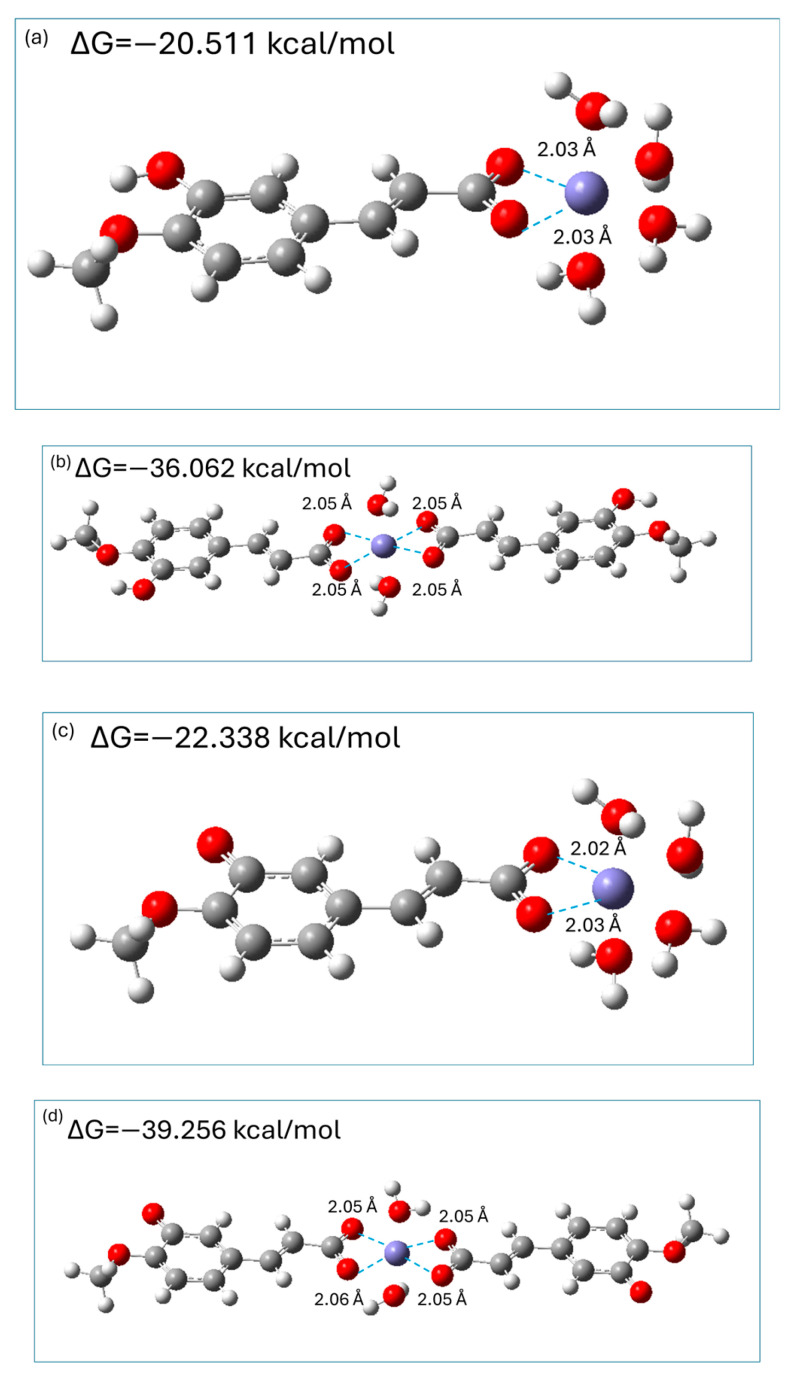
M062X/6-311+G(d,p)/PCM optimized geometries of 1:1 (Fe^2+^/monoanion (**a**); Fe^2+^/dianion (**c**)) and 1:2 ((Fe^2+^/monoanion (**b**); Fe^2+^/dianion (**d**)) complexes.

It is generally accepted [3,39,40,41] that Fe^2+^ coordinates six electron pair donor atoms of ligand(s) in an octahedral geometry; therefore, in the aqueous phase, hydrated [Fe(H_2_O)_6_]^2+^ ions exist. Previous theoretical studies [3,40,41] on the chelation of metal ions by hydroxycinnamic acids revealed that the carboxylic moiety is the preferable coordination site; therefore, both monoanionic and dianionic species of isoferulic acid were considered as bidentate ligands which may form 1:1 and 1:2 complexes with hydrated Fe^2+^ ions. M062X/6-311+G(d,p)/PCM optimized geometries of 1:1 and 1:2 complexes of Fe^2+^: monoanionic (dianionic) species of isoferulic acid are presented in Figure 9 together with the calculated Gibbs free energies ΔG for the corresponding chelation reactions.

The results of calculations indicated that the resultant complexes are stable chelates where bidentate isoferulate ligand(s) replace two (1:1 chelates) or four (1:2 chelates) water molecules forming stable complexes in which the distance between the carboxylic oxygen atoms and Fe^2+^ ions is 2 Å (Figure 8). The chelation reactions are exergonic (see Figure 8), indicating that they are spontaneous. Moreover, the complexes with 1:2 metal-to-ligand molar ratios are almost twice as stable as the corresponding 1:1 complexes. In addition, the 1:1 and 1:2 complexes with dianionic species are of 2 kcal/mol and 3 kcal/mol, respectively, more stable as compared to those with the monoanionic form. Thus, dianionic species of isoferulic acid chelate Fe^2+^ ions via a carboxylic moiety only slightly more effectively than their monoanionic counterparts.

The M062X/6-311+G(d,p)/PCM-predicted Gibbs free energies of the complexation of hydrated Fe^2+^ by the monoanionic form of isoferulic acid are similar to those previously obtained by Truong et al. at the M05/6-311++G(d,p)/SMD level [41] for ferulic acid (approximately −22 kcal/mol and −45 kcal/mol for 1:1 and 1:2 metal–ligand complexes, respectively) and are more exergonic compared to the values reported by Amić [40] using the M06/6-311++G(d,p)/SMD method.

The obtained results showed that at physiological pH, isoferulic acid acts as an effective Fe^2+^ chelator; thus, it may be classified into the category of type II antioxidant. Another mode of antioxidant action, which is characteristic of type II antioxidants, is their ability to absorb UV electromagnetic radiation. Exposure to UVB and UVA radiation may lead to the excessive formation of free radicals and, as a result, to oxidative stress. A necessary condition for an antioxidant chromophore to exhibit such secondary antioxidant activity is that its absorption spectrum must fall within the UV wavelength range of electromagnetic radiation.

### 2.8. Theoretical Prediction of Absorption Spectra of Isoferulic Acid in Aqueous Medium at Different pH

**Figure 10:**
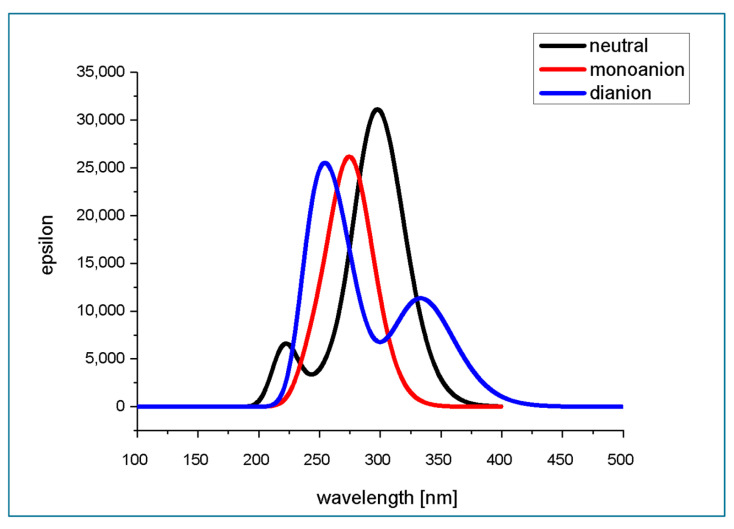
TD M062X/6-311+G(d,p)/PCM(water)-predicted absorption spectra of neutral and deprotonated forms of isoferulic acid.

TD M062X/6-311+G(d,p)/PCM(water)-predicted absorption spectra of neutral and deprotonated forms of isoferulic acid are presented in Figure 10, while the calculated parameters of electronic transitions to the six lowest singlet states are gathered in Table 11. The theoretically predicted absorption spectra of isoferulic acid in aqueous solution at various pH values (Figure 10) are quite consistent with previously reported experimental spectra for its isomer, ferulic acid [31].

The theoretically predicted absorption spectrum of the neutral form of isoferulic acid in water consists of an intense band with a maximum at ~300 nm and a less intense shorter-wavelength band with a maximum located at ~225 nm. The experimental maxima of isoferulic acid absorption spectrum in methanol are reported to be located at 243 nm (5.10 eV), 294 nm (4.22 eV), and 322 nm (3.85 eV) [18]. Taking into account that the typical error for TD-DFT determined that electronic excitation energy falls within the range 0.1–0.5 eV and is almost comparable with that of high-level correlated theoretical approaches [42], and considering that the solvent is expected to affect the location of the absorption band maxima, the theoretically predicted absorption spectrum of the neutral form of isoferulic acid is in satisfactory agreement with the experimental data. The longer-wavelength absorption band of the neutral form of isoferulic acid arises from the intense (f = 0.7577) S_0_ → S_1_ electronic transition of the energy of 4.16 eV (298.29 nm) (see Table 11). Analysis of the molecular orbital contribution involved in this electronic transition revealed that it is a HOMO → LUMO transition (which, looking at the molecular orbitals’ graphical representation (Figure 3) occurs between states of π and π* character). A less intense (f = 0.0926) electronic transition of the energy of 4.74 eV (261.44 nm) is obscured under the former transition. The less intense absorption band of the neutral form of isoferulic acid, located at shorter wavelengths of the spectrum with a maximum at ~ 225 nm, originates from a relatively intense (f = 0.1608) electronic transition of the energy of 5.58 eV (222.22 nm) and a less intense (f = 0.0130) electronic transition of the energy of 5.62 eV (220.44 nm).

The excitation energies and oscillator strengths determined for the three lowest electronic transitions of neutral isoferulic acid (Table 11) are comparable to those reported by Mazzone et al. [38] for the neutral form of ferulic acid in ethanol with the use of the M052X/6-31+G** (S_0_–S_1_: 4.10 eV, f = 0.709; S_0_–S_2_: 4.82 eV, f = 0.055; S_0_–S_3_: 5.61 eV, f = 0.640) and wB97XD/6-31+G** methods (S_0_–S_1_: 4.07 eV, f = 0.701; S_0_–S_2_: 4.70 eV, f = 0.054; S_0_–S_3_: 5.53 eV, f = 0.548).

The theoretically predicted absorption spectrum of the monoanionic form of isoferulic acid in water is characterized by a single band with a maximum at ~ 275 nm which mainly arises from the intense (f = 0.6103) π → π* electronic transition of the energy of 4.48 eV (276.48 nm) between the HOMO and the LUMO. A less intense (f = 0.1708) electronic transition of the energy 5.00 eV (248.08 nm) also contributes to this band.

In the UV absorption spectrum of dianionic forms of isoferulic acid in water, two absorption bands may be seen: the shorter-wavelength band with a maximum at ~260 nm and a less intense longer-wavelength band with a maximum at 340 nm. The longer-wavelength absorption band arises only from the HOMO → LUMO (of a π → π* type) electronic transition of the energy of 3.71 eV (333.81 nm) and oscillator strength of 0.2783, while two strong electronic transitions of the energy of 4.63 eV (267.54 nm; f = 0.3373) and 5.02 eV (247.13 nm; f = 0.4483) contribute to the higher energy band of the spectrum.

From Figure 10, it may be seen that isoferulic acid exhibits strong absorption of electromagnetic radiation within the UVB (290–320 nm) wavelength range. Moreover, dianionic species of isoferulic acid also absorb within the UVA (320–400 nm) wavelength range.

Although this study investigates several antioxidant mechanisms of isoferulic acid, including radical scavenging, UV absorption, and metal chelation, it does not cover some important aspects such as its ability to inhibit prooxidant enzymes like lipoxygenase (LOX), which catalyze the generation of reactive oxygen species [40]. Furthermore, its potential to scavenge reactive nitrogen species (RNS) and reactive sulfur species (RSS), as well as its antioxidant activity in lipid environments, remains unexplored. These aspects deserve further investigation in future studies.

## 3. Materials and Methods

### 3.1. Electronic and Geometrical Structure of Isoferulic Acid

Geometric optimization and subsequent frequency calculations of the molecular structures of H_2_O_2_ and isoferulic acid (in neutral, monoanionic and dianionic forms) and corresponding radicals were performed at the M062X/6-311+G(d,p)/PCM(water) level of theory. This level of theory is a compromise between theoretical outcomes and computational resource uptake. For closed-shell systems, restricted computations were performed, while for open-shell systems (radicals), unrestricted calculation schemes were applied. Analysis of the calculated frequencies was performed to confirm the nature of the obtained stationary points; no imaginary frequencies were obtained for local minima, whereas transition states were characterized by only one imaginary frequency. All calculations were performed with the use of Gaussian 09 [43] and the GaussView 5.0 suite of programs [44].

### 3.2. Deprotonation Constants

The pK_a_ values for two-step isoferulic acid deprotonation were calculated using two methods: the relative (isodesmic) method and the parameter-fitting method [8,45]. The free energy of solvated species was calculated with the use of the M062X/6-311+G(d,p)/PCM(water) method.

#### 3.2.1. Isodesmic Method

The isodesmic method relies on the proton exchange equilibrium between the acid H_2_A and the conjugated base of the reference acid (Ref), according to the following reactions:

(1) H_2_A + HA^−^(Ref) → HA^−^ + H_2_A (Ref);
(2) HA^−^ + A^2−^(Ref) → A^2−^ + HA^−^ (Ref).

In the pK_a_ determination of isoferulic acid, 3-hydroxy-4-methoxybenzoic acid (isovanillic acid) was taken as the reference compound (with pK_a_ values: pK_a1_ = 4.3 and pK_a2_ = 9.5 taken from [30]).

The pK_a_ values corresponding to all of the deprotonation steps (pK_a1_, pK_a2_) of isoferulic acid were calculated from the following equation:

$${pK}_{a}=\frac{{\Delta G}_{s}^{*}}{2.303\cdot RT}+pK_{a}\left(Ref\right)$$

where ${\Delta G}_{s}^{*}$ is the free energy of the appropriate deprotonation reaction [8], calculated as the difference between the free energy of solvated products and reactants [J/mol], R is the gas constant [J/molK], and T is the standard temperature [K].

#### 3.2.2. Parameter-Fitting Method

pK_a_ values corresponding to each of the deprotonation steps (pK_a1_, pK_a2_) of isoferulic acid were calculated at the M062X/6-311+G(d,p)/PCM(water) level of theory using a previously reported [45] parameter-fitting methodology. These values were used to calculate the molar fractions of neutral and deprotonated species of isoferulic acid at different pH values.

### 3.3. Indices Related to Frontier Molecular Orbitals Theory

Indices related to Frontier Molecular Orbitals Theory for isoferulic acid were calculated using the M062X/6-311+G(d,p)/PCM(water) method. The ionization potential (IP) and electron affinity (EA) of the isolated species were evaluated according to Janak’s theorem [46,47].

Having these values, descriptions of indices related to Frontier Molecular Orbitals Theory—electronegativity (χ), chemical potential (μ), global hardness (η), global softness (S), and electrophilicity (ω), as well as electron-donating power (ω−) and electron-accepting power (ω+)—were calculated [8,48,49,50].

### 3.4. Prediction of Radical Attack Site Based on Condensed Fukui Functions

Electron population analysis (at the M062X/6-311+G(d,p)/PCM level of theory) was performed with the use of natural bonding orbitals (NBOs). The obtained atomic charge values of (N − 1) and (N + 1) systems were used to calculate condensed Fukui indices for each atom A of the isoferulic acid molecule (dianionic form) according to the following equation [51]:

$$f_{A}^{0}=\frac{\left(q_{N-1}^{A}-q_{N+1}^{A}\right)}{2}$$

where $f_{A}^{0}$ denotes the Fukui index for the free radical attack of atom A; and $q_{N-1}^{A}$ and $q_{N+1}^{A}$ denote the atomic charge of atom A in a molecule with (N − 1) and (N + 1) electrons, respectively, obtained vertically from the optimized ground-state geometry of a molecule with N electrons.

### 3.5. Intrinsic Thermochemical Reactivity Indices

The intrinsic thermochemical reactivity indices of bond dissociation enthalpy (BDE), adiabatic ionization potential (AIP), proton affinity (PA), and proton dissociation enthalpy (PDE) were calculated at the M062X/6-311+G(d,p)/PCM(water) level of theory using the previously described methodology [8,52]. The enthalpies of the solvated proton (−1052.7 kJ/mol) and electron (−98.8 kJ/mol) were taken from [53].

### 3.6. Thermodynamics of HOO^●^ Free Radical Scavenging Reaction Pathways

The Gibbs free energies of the reactions involved in HT (hydrogen transfer), RAF (radical adduct formation), SET (single electron transfer), SET-PT (sequential electron transfer–proton transfer), SPL-ET (sequential proton loss–electron transfer), and SPL-HT (sequential proton loss–hydrogen transfer) were calculated at the M062X/6-311+G(d,p)/PCM(water) level of theory applying the previously described methodology [6,8,52]. The Gibbs free energies of the reactions involved in HT (hydrogen transfer) and RAF (radical adduct formation) were calculated in the framework of the 1 M standard state [7].

### 3.7. Rate Constants for HT and RAF Reactions

In the HT and RAF mechanisms, TS is encountered along the reaction pathways. It was assumed that in the HT and RAF mechanisms, the two respective reactants (antioxidant and hydroperoxyl radical) form the reactant complex RC and the process is considered as a unimolecular reaction of the RC [6,7,8,14]. Then, bimolecular rate constants for the reactions involved in the HT and RAF pathways were calculated (at the M062X/6-311+G(d,p)/PCM(water) level) in the framework of conventional transition state theory (taking into account tunneling phenomena and reaction path degeneracy) and the 1 M standard state using the following equation [6,7,8,54,55]:

$$k_{bim}=\sigma \kappa \frac{k_{B}T}{h}e^{-(∆G_{a}^{\#})/RT}$$

where σ and κ represent the number of equivalent reaction pathways (i.e., reaction path degeneracy) [56,57] and Eckart tunneling correction [58], respectively; $k_{B}$—Boltzmann constant, h—Planck constant, T—standard temperature, R—gas constant, $∆G_{a}^{\#}$—free activation energy; and the Eyringpy program is used [59,60].

In order to find and optimize transition state structures in the HT and RAF reaction pathways, the method implemented by Schlegel and coworkers [61,62] was applied (requested by the QST3 option in Gaussian 09). Then, for the identified TS, the intrinsic reaction coordinate (IRC) was constructed starting from the respective TS geometry and going downhill to both the reactant and the product channels to ensure that the obtained TS corresponds to the reactions involved in the HT and RAF mechanisms.

### 3.8. Rate Constant for Single Electron Transfer (SET) Reaction

In the SET reaction, there is no transition state (TS) between reactants and products. The energy barrier for electron transfer (SET mechanism) was calculated on the basis of the Marcus theory [63,64], according to the following equation:

$${\Delta G}_{a}^{\#}=\frac{\lambda }{4}{\left(1+\frac{∆G}{\lambda }\right)}^{2}$$

where λ is the nuclear reorganization energy, calculated as the difference between ΔE (the vertical energy between reactants and products of the reaction via SET mechanism) and the adiabatic free energy of the reaction ∆G:

λ=ΔE−∆G

The bimolecular rate constant for the SET reaction (k_bim_) was calculated in the framework of the 1 M standard state [7] using the following equation [54]:

$$k_{bim}=\frac{k_{B}T}{h}exp\left(\frac{-{\Delta G}_{a}^{\#}}{RT}\right)$$

where k_B_—Boltzmann constant, h—Planck constant, R—gas constant, T—absolute temperature.

### 3.9. Correction for Diffusion-Controlled Rates

For bimolecular rate constants close to the diffusion limit, the apparent rate constants k_app_ were calculated based on the Collins–Kimball theory [65]:

$$k_{app}=\frac{k_{D}k_{bim}}{k_{D}+k_{bim}}$$

where k_D_ is the rate constant for an irreversible bimolecular diffusion-controlled reaction calculated following Smoluchowski [66]:

$$k_{D}=4\pi R_{AB}D_{AB}N_{A}$$

where N_A_ is the Avogadro number; $R_{AB}$ is the distance at which the reaction takes place, calculated as the sum of the reactants’ radii; and $D_{AB}$ is the mutual diffusion coefficient of antioxidant A and radical B calculated using the Stokes–Einstein approach [67,68] as the sum of the corresponding diffusion coefficients:

$$D_{A}=\frac{k_{B}T}{{6\pi \eta R}_{A}}$$

$$D_{B}=\frac{k_{B}T}{{6\pi \eta R}_{B}}$$

where η is the viscosity of water (0.8905 × 10^−3^ Pa·s [69]).

### 3.10. Chelation Ability of Isoferulic Acid

The Gibbs free energies of the following chelation reactions, leading to the formation of complexes between monoanionic and dianionic species of isoferulic acid and hydrated Fe^2+^ ions with 1:1 and 1:2 metal-to-ligand molar ratios,

[Fe(H_2_O)_6_]^2+^ + HA^−^ → [(HA)Fe(H_2_O)_4_]^+^ + 2 H_2_O

[Fe(H_2_O)_6_]^2+^ + A^2−^ → [(A)Fe(H_2_O)_4_] + 2 H_2_O

[Fe(H_2_O)_6_]^2+^ + 2 HA^−^ → [(HA)_2_Fe(H_2_O)_2_] + 4 H_2_O

[Fe(H_2_O)_6_]^2+^ + 2 A^2−^ → [(A)_2_Fe(H_2_O)_2_]^2−^ + 4 H_2_O

were calculated at the M062X/6-311+G(d,p)/PCM(water) level.

### 3.11. Theoretical Prediction of Absorption Spectra and Parameters of Electronic Transitions to the Excited Singlet States

The parameters of electronic vertical transitions to the excited singlet states (oscillator strengths, excitation energies [eV] with their corresponding wavelengths [nm], ground to excited state transition electric dipole moments [D]) of the neutral and deprotonated forms of isoferulic acid were calculated with the use of TD-M062X/6-311+G(d,p)/PCM(water). The absorption spectra of the studied compounds were calculated based on vertical excitations involving the six lowest-energy excited states.

## 4. Conclusions

The obtained results of M062X/6-311+G(d,p)/PCM(water) calculations performed in the framework of this work indicate that at physiological pH (7.4; where monoanionic species formed as a result of carboxylic group deprotonation are the dominant acid–base species), isoferulic acid exhibits primary (via the RAF and HT mechanism) antioxidant activity against HOO^•^ radicals. Additionally, deprotonated species of isoferulic acid effectively chelate hydrated Fe^2+^ ions, forming stable complexes and thereby entrapping catalytic transition metal ions, which prevents the generation of highly harmful and reactive hydroxyl radicals in the Fenton reaction. Another mode of secondary antioxidant activity of isoferulic acid is via the strong absorption of electromagnetic radiation within the UV range, which additionally contributes to the limitation of the photo-induced generation of free radicals. The thermodynamic and kinetic data collected in this study showed that the RAF pathway is a more preferable pathway of hydroperoxyl radical scavenging of isoferulic acid as compared to HT, since the radical adduct formation reaction is more exergonic and eight-times faster than the reaction involved in the HT mechanism. Inspection of the optimized geometries of RAF products revealed that both intramolecular and intermolecular hydrogen-bonding interactions contribute to the stability of the obtained adducts and their spin density is spread over the entire molecule resulting in significant radical stabilization. Moreover, the calculated temperature-dependent transmission coefficient showed the importance of including a tunneling effect in kinetic calculations for the HT mechanism. Inspection of the geometrical parameters of stationary points encountered along the HT pathway showed that hydrogen-bonding interactions provide a favorable geometrical rearrangement facilitating hydrogen transfer.

In an acidic medium (pH < pK_a1_), where neutral forms of isoferulic acid are the dominant acid–base species, the compound under study exhibits a lower ability to scavenge hydroperoxyl radicals, mainly via the HT mechanism. Increasing the pH of the medium to values greater than the pK_a2_ of isoferulic acid (where phenolate dianionic species prevail) boosts the antioxidant activity of the antioxidant studied by the occurrence of fast, limited only by diffusion electron-related SET channel. The results of my calculations showed that the electron-donating ability of isoferulic acid phenolate ions is high enough to promote a fast electron transfer mechanism towards not only hydroperoxyl radicals but also towards other electrophilic free radicals.

Due to its antioxidant properties and the ability to absorb electromagnetic radiation within the UVB range, isoferulic acid and/or plant extracts rich in this phytochemical may be considered as a potential candidate for the photoprotective ingredient of sunscreen cosmetics formulations.

The theoretical insights presented in this study not only clarify the multi-target antioxidant behavior of isoferulic acid, highlighting its promising role as a photoprotective agent in sunscreen formulations, but also extend our knowledge on the free radical scavenging mechanisms of structurally related phenolic compounds.

## Figures and Tables

**Table 1:** M062X/6-311+G(d,p)/PCM (water) values of pK_a_ for isoferulic acid determined with the use of isodesmic and parameter-fitting methods.

pK_a_	Isodesmic Method *	Parameter-Fitting Method	Literature Predicted Value [29]
pK_a1_	4.10	5.30	4.53 ± 0.11
pK_a2_	6.30	10.80	-

* pK_a_ values of reference compound 3-hydroxy-4-methoxybenzoic acid (isovanillic acid): pK_a1_ = 4.3; pK_a2_ = 9.5, were taken from [30].

**Table 2:** M062X/6-311+G(d,p)/PCM(water)-calculated indices related to Frontier Molecular Orbitals Theory.

Isoferulic Acid	IP [kcal/mol]	EA [kcal/mol]	Χ [kcal/mol]	μ [kcal/mol]	Ƞ [kcal/mol]	S [kcal/mol	ω [kcal/mol]	ω− [kcal/mol]	ω+ [kcal/mol]
neutral	172.00	28.96	100.48	−100.48	71.52	35.76	70.58	129.76	17.65
monoanion	164.30	10.63	87.47	−87.47	76.84	38.42	49.78	103.12	12.45
dianion	133.04	0.93	66.99	−66.99	66.06	33.03	33.96	75.72	8.49

**Table 3:** The condensed Fukui indices $f_{A}^{0}$ for O and C atoms in dianionic species of isoferulic acid.

Atom	Dianion
O_1_	0.034
O_2_	0.043
O_3_	0.039
O_21_	0.170
C_4_	0.007
C_5_	0.109
C_6_	0.125
C_7_	0.000
C_8_	0.132
C_9_	−0.007
C_10_	0.076
C_11_	0.143
C_12_	−0.006
C_13_	0.010

Atomic labels according to Gaussian 09—see Figure 2.

**Table 4:** M062X/6-311+G(d,p)/PCM(water)-calculated values of adiabatic IP (AIP), BDE, PA, and PDE.

Isoferulic Acid	AIP [kcal/mol]	BDE [kcal/mol]	PA [kcal/mol]	PDE [kcal/mol]
neutral	118.53	86.34	31.60	3.71
monoanion	110.98	84.70	42.74	9.63
dianion	77.87	106.78	-	64.83

AIP—adiabatic ionization potential, BDE—bond dissociation energy, PA—proton affinity, PDE—proton dissociation enthalpy.

**Table 5:** M062X/6-311+G(d,p)/PCM(water)-calculated values of EA for selected radicals.

Radical	EA * [kcal/mol]
HOO^•^	−71.858
NO_2_^•^	−96.043
HO^•^	−93.008
SO_4_^•−^	−114.48
CH_3_O^•^	−78.429
Cl_3_CO^•^	−142.315

* EA was calculated as the enthalpy change accompanying the reaction R^●^ + e^−^ = R^−^.

**Table 6:** M062X/6-311+G(d,p)/PCM(water) Gibbs free energies [kcal/mol] at 298.15 of the reactions involved in the HT, SET, SET-PT, SPL-ET, and SPL-HT antioxidant mechanisms of isoferulic acid towards HOO^●^ radicals.

Isoferulic Acid	RAF ΔG [kcal/mol]	HT ΔG [kcal/mol]	SET ΔG [kcal/mol]	SET-PT ΔG [kcal/mol]	SPL-ET ΔG [kcal/mol]	SPL-HT ΔG [kcal/mol]
neutral	+2.858 (C_11_)	−2.332 (O_21_)	+44.126	(1) 44.126 (2) −48.001 (O_21_)	(1) −32.093 * (2) +36.649	(1) −32.093 * (2) −3.894
monoanion	−6.245 (C_11_)	−3.894 (O_21_)	+36.649	(1) 36.649 (2) −42.086 (O_21_)	(1) −20.646 (2) +3.793	(1) −20.646 (2) +17.776 (C_11_)
dianion	−5.801 (C_11_)	+17.776 (C_11_)	+3.793	(1) +3.793 (2) +12.440	-	-

* monoanion. Atomic labels according to Gaussian 09—see Figure 4.

**Table 7:** M062X/6-311G+(d,p)/PCM(water)-calculated geometrical parameters of the most stable products of the RAF reaction incorporating neutral and deprotonated species of isoferulic with HOO^●^ radicals.

Bond Length [Å]	Neutral	Monoanionic	Dianionic
C_4_C_10_	1.41	1.41	1.42
C_10_C_11_	1.49	1.49	1.49
C_11_C_12_	1.53	1.56	1.56
C_12_O_2_	1.33	1.24	1.24
C_12_O_3_	1.20	1.26	1.26
C_11_C_18_	1.42	1.43	1.44
O_18_O_17_	1.42	1.43	1.43
O_2_H_16_	2.59	2.57	2.58
**O_3_H_19_**	**2.10**	**1.68**	**1.68**
O_1_H_26_	2.09	2.09	-
bond angle [°]			
O_1_H_26_O_25_	113.7	114.1	-
C_4_C_10_C_11_	124.9	125.4	125.9
C_10_C_11_C_12_	112.1	112.2	111.9
C_11_C_12_O_2_	111.5	116.0	116.4
C_11_C_12_O_3_	124.2	116.3	116.6
C_11_H_16_O_2_	65.9	68.0	67.5
C_11_O_18_O_17_	109.6	109.3	109.5
C_10_C_11_H_16_	111.5	111.3	111.3
C_10_C_11_O_18_	113.2	111.4	112.1
**O_17_H_19_O_3_**	**123.5**	**145.4**	**145.9**
dihedral angle [°]			
C_4_C_10_C_11_C_12_	91.8	87.3	100.2
C_6_C_4_C_10_C_11_	−1.06	−2.40	−0.59
C_10_C_11_C_12_O_2_	−64.5	−74.9	−75.9
C_4_C_10_C_11_O_18_	−144.2	−146.8	−134.1
C_10_C_11_O_18_O_17_	−57.6	−63.4	−62.8

Geometrical parameters of hydrogen-bonded interactions are marked with bold font; atomic labels according to Gaussian 09.

**Table 8:** The calculated relative enthalpy H [kJ/mol] and relative free energy values G [kJ/mol] of the stationary points along the reaction coordinate in the HT and RAF mechanisms. The values were calculated with respect to those for isolated reagents.

	RC		TS		PC		Products	
	H [kJ/mol]	G [kJ/mol]	H [kJ/mol]	G [kJ/mol]	H [kJ/mol]	G [kJ/mol]	H [kJ/mol]	G [kJ/mol]
HT	−25.496	6.785	39.750	80.834	−20.285	15.220	−6.934	−16.291
RAF	−72.264	−36.203	11.476	56.210	−73.535	−25.877	−73.643	−26.131

**Table 9:** Kinetic parameters (Gibbs free energies of activation (ΔG_a_^#^), Eckart transmission coefficient (κ), diffusion rate constants (k_D_), bimolecular rate constant (k_bim_), and diffusion-corrected apparent rate constants (k_app_)) of the reactions involved in the HT and RAF antioxidant mechanisms (for monoanionic species of isoferulic acid) towards HOO^●^.

pH 7 298.15 K	ΔG_a_^#^ [kcal/mol]	κ	k_D_ [M^−1^s^−1^]	k_bim_ [M^−1^s^−1^]	k_app_ [M^−1^s^−1^]
HT	19.3	3230.5	2.0 × 10^9^	5.6 × 10^2^	5.6 × 10^2^
RAF	13.4	1.7	2.0 × 10^9^	4.5 × 10^3^	4.5 × 10^3^

**Table 10:** Kinetic parameters (reaction Gibbs free energies (ΔG), Gibbs free energies of activation (ΔG_a_^#^), reorganization energies (λ), rate constants (k_bim_), diffusion rate constants (k_D_), and diffusion-corrected apparent rate constants (k_app_)) of the reactions involved in the SET antioxidant mechanism of the dianionic form of isoferulic acid towards various radicals.

Radical	ΔG [kJ/mol]	ΔG_a_^#^ [kJ/mol]	λ [kJ/mol]	k_bim_ [M^−1^s^−1^]	k_D_ [M^−1^s^−1^]	k_app_ [M^−1^s^−1^]
HOO^•^	15.871	20.591	45.028	1.53 × 10^9^	7.84 × 10^6^	7.80 × 10^6^
NO_2_^•^	−85.392	15.325	194.614	1.28 × 10^10^	7.89 × 10^6^	7.88 × 10^6^
HO^•^	−71.833	0.706	87.552	4.67 × 10^12^	8.14 × 10^6^	8.14 × 10^6^
SO_4_^•−^	−153.662	6.925	234.210	3.80 × 10^11^	7.57 × 10^6^	7.57 × 10^6^
CH_3_O^•^	−10.707	4.847	37.767	8.79 × 10^11^	7.83 × 10^6^	7.83 × 10^6^
Cl_3_CO^•^	−281.495	6.751	383.224	4.08 × 10^11^	7.51 × 10^6^	7.51 × 10^6^

**Table 11:** TD M062X/6-311+G(d,p)/PCM(water)-calculated parameters (excitation energies (eV) and their corresponding wavelengths (λ), oscillator strengths (f), electric transition dipole moments (μ)) of electronic transitions to the six lowest singlet excited states of isoferulic acid.

	Neutral			Monoanion				Dianion	
	λ [nm] (eV)	f	μ [D]	λ [nm] (eV)	f	μ [D]	λ [nm] (eV)	f	μ [D]
1	298.29 (4.16)	0.7577	7.4406	276.48 (4.48)	0.6103	5.5546	333.81 (3.71)	0.2783	3.0585
2	261.44 (4.74)	0.0926	0.7967	257.95 (4.81)	0.0000	0.0003	292.73 (4.24)	0.0007	0.0064
3	241.20 (5.14)	0.0000	0.0002	254.03 (4.88)	0.0000	0.0000	267.54 (4.63)	0.3373	2.9712
4	222.22 (5.58)	0.1608	1.1760	248.08 (5.00)	0.1708	1.3945	262.67 (4.72)	0.0005	0.0047
5	220.44 (5.62)	0.0130	0.0097	229.75 (5.40)	0.0016	0.0119	253.41 (4.89)	0.0000	0.0004
6	203.52 (6.09)	0.0004	0.0026	212.39 (5.84)	0.0005	0.0034	247.13 (5.02)	0.4482	3.6466

## Data Availability

Data are contained within the article.
